# Supplementary material for: Flavonoids from Sedum japonicum subsp. oryzifolium (Crassulaceae)
Source: Molecules. 2022 Nov 7;27(21):7632. doi: 10.3390/molecules27217632 (PMC9656034; doi:10.3390/molecules27217632)
Supplement: Supplementary file 1 [file molecules-27-07632-s001.zip › molecules-1996644-supplementary.pdf]

Supplementary data

Flavonoids from *Sedum japonicum* subsp. *oryzifolium* (Crassulaceae)

Takayuki Mizuno[1], Nahoko Uchiyama[2], Seiji Tanaka[2], Takahisa Nakane[3], Kazumi Fujikawa[4], Nobuo Kawahara[4] and Tsukasa Iwashina[1,*]

[1]Department of Botany, National Museum of Nature and Science, 4-1-1 Amakubo, Tsukuba 305-0005, Japan; iwashina@kahaku.go.jp (T.I.); tmizuno@kahaku.go.jp (T.M.)

[2]Division of Pharmacognosy, Phytochemistry and Narcotics/National Institute of Health Science (NHS), 3-25-26 Tonomachi, Kawasaki-ku, Kawasaki, Kanagawa 210-9501, Japan; nuchiyama@nihs.go.jp (N.U.); seiji-tanaka@nihs.go.jp (S.T.)

[3]Showa Pharmaceutical University, 3-3165 Higashi-tamagawagakuen, Machida, Tokyo 194-8543, Japan; nakane@ac.shoyaku.ac.jp (T.N.)

[4]The Kochi Prefectural Makino Botanical Garden, 4200-6 Godaisan, Kochi 781-8125, Japan; saussure@makino.or.jp (K.F.); kawahara@makino.or.jp (N.K.)

Contents of Figures

Figure 1-1S. 800 MHz ^{1}H NMR spectrum of **3** in DMSO-d_6
Figure 1-2S. 200 MHz ^{13}C NMR spectrum of **3** in DMSO-d_6
Figure 1-3S. 800 MHz HMQC spectra of **3** in DMSO-d_6
Figure 1-4S. 800 MHz HMBC spectrum of **3** in DMSO-d_6
Figure 1-5S. 800 MHz COSY spectrum of **3** in DMSO-d_6
Figure 1-6S. 800 MHz NOESY spectrum of **3** in DMSO-d_6
Figure 2-1S. 800 MHz ^{1}H NMR spectrum of **4** in DMSO-d_6
Figure 2-2S. 200 MHz ^{13}C NMR spectrum of **4** in DMSO-d_6
Figure 2-3S. 800 MHz HMQC spectra of **4** in DMSO-d_6
Figure 2-4S. 800 MHz HMBC spectrum of **4** in DMSO-d_6
Figure 2-5S. 800 MHz COSY spectrum of **4** in DMSO-d_6
Figure 3-1S. 800 MHz ^{1}H NMR spectrum of **6** in DMSO-d_6
Figure 3-2S. 200 MHz ^{13}C NMR spectrum of **6** in DMSO-d_6
Figure 3-3S. 800 MHz HMQC spectra of **6** in DMSO-d_6
Figure 3-4S. 800 MHz HMBC spectrum of **6** in DMSO-d_6
Figure 3-5S. 800 MHz COSY spectrum of **6** in DMSO-d_6
Figure 3-6S. 800 MHz NOESY spectrum of **6** in DMSO-d_6
Figure 4-1S. 800 MHz ^{1}H NMR spectrum of **7** in DMSO-d_6

Figure 4-2S. 200 MHz ^{13}C NMR spectrum of **7** in DMSO-d_6 Fig. 4-3S. 800 MHz HMQC spectra of **7** in DMSO-d_6 Fig. 4-4S. 800 MHz HMBC spectrum of **7** in DMSO-d_6 Fig. 4-5S. 800 MHz COSY spectrum of **7** in DMSO-d_6 Fig. 5-1S. 800 MHz ^{1}H NMR spectrum of **8** in DMSO-d_6 Fig. 5-2S. 200 MHz ^{13}C NMR spectrum of **8** in DMSO-d_6 Fig. 5-3S. 800 MHz HMQC spectra of **8** in DMSO-d_6 Fig. 5-4S. 800 MHz HMBC spectrum of **8** in DMSO-d_6 Fig. 5-5S. 800 MHz COSY spectrum of **8** in DMSO-d_6 Fig. 5-6S. 800 MHz NOESY spectrum of **8** in DMSO-d_6

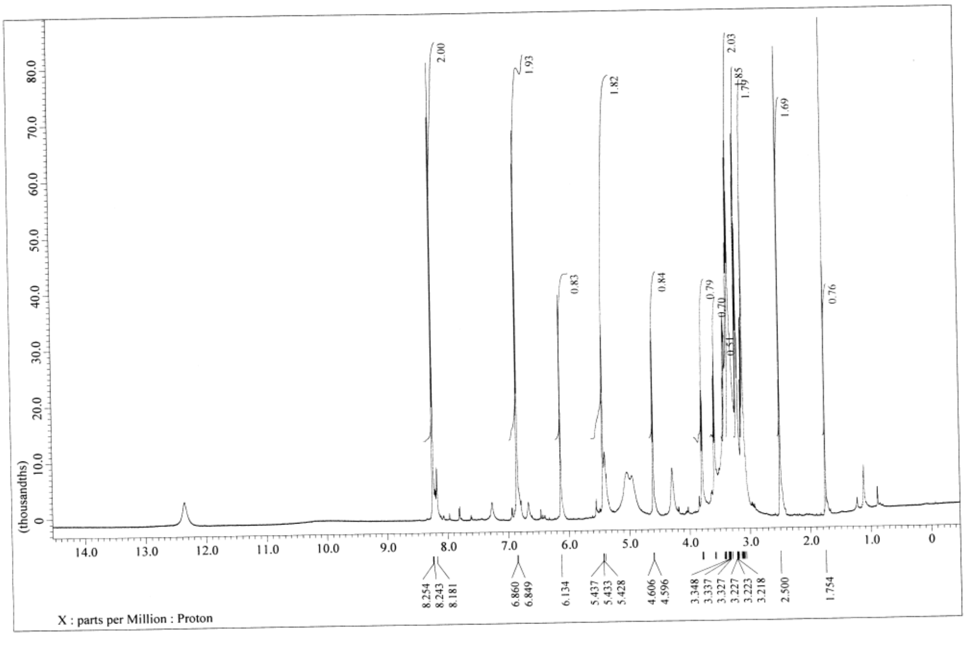

Figure 1-1S. 800 MHz ^{1}H NMR spectrum of **3** in DMSO-d_6.

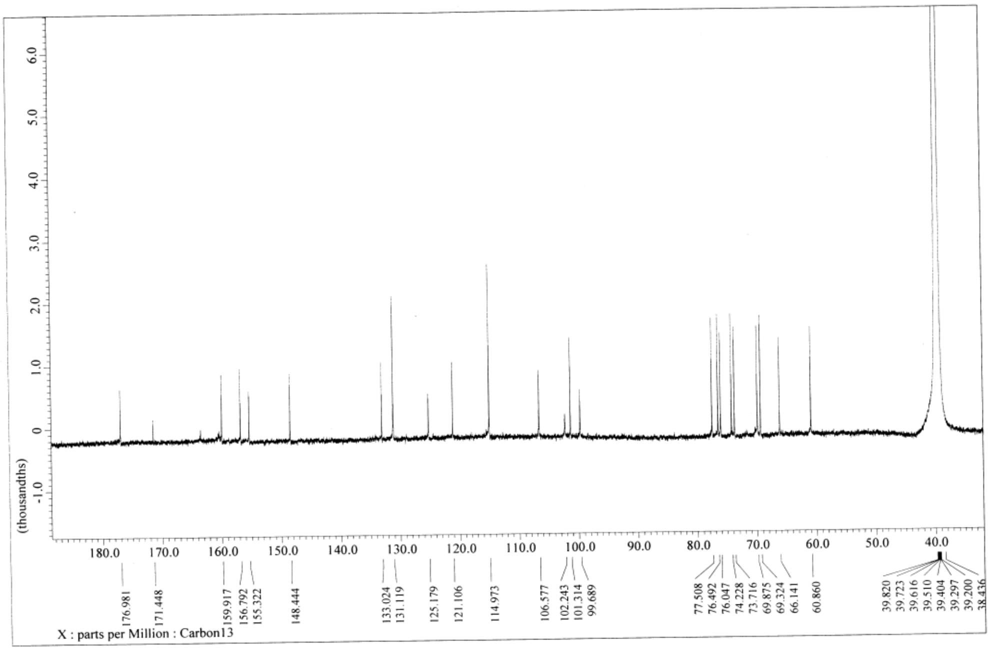

Figure 1-2S. 200 MHz ^{13}C NMR spectrum of **3** in DMSO-d_6

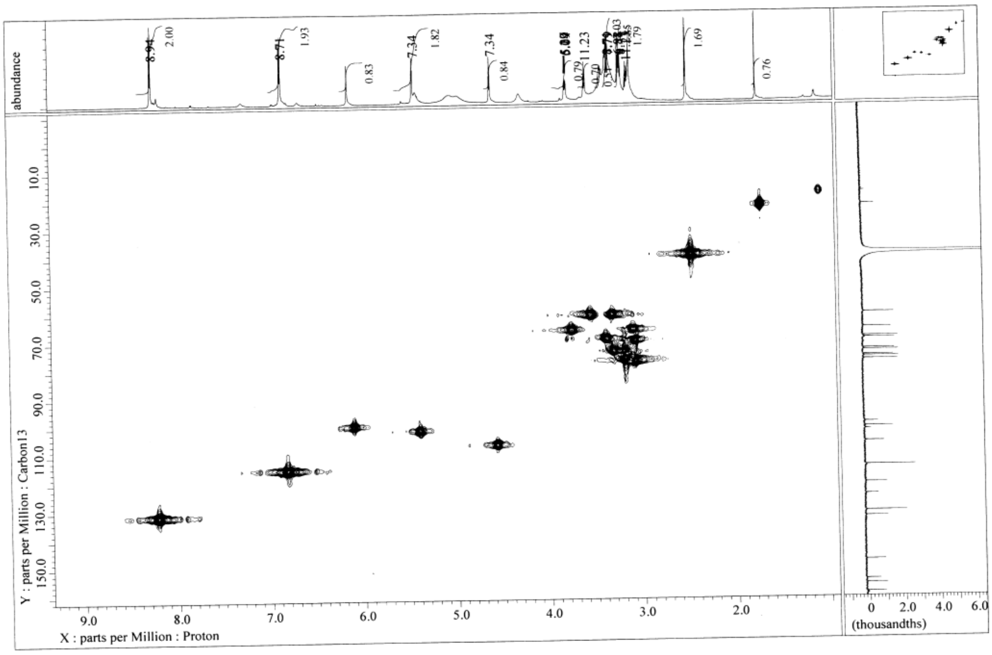

Figure 1-3S. 800 MHz HMQC spectra of **3** in DMSO-d_6

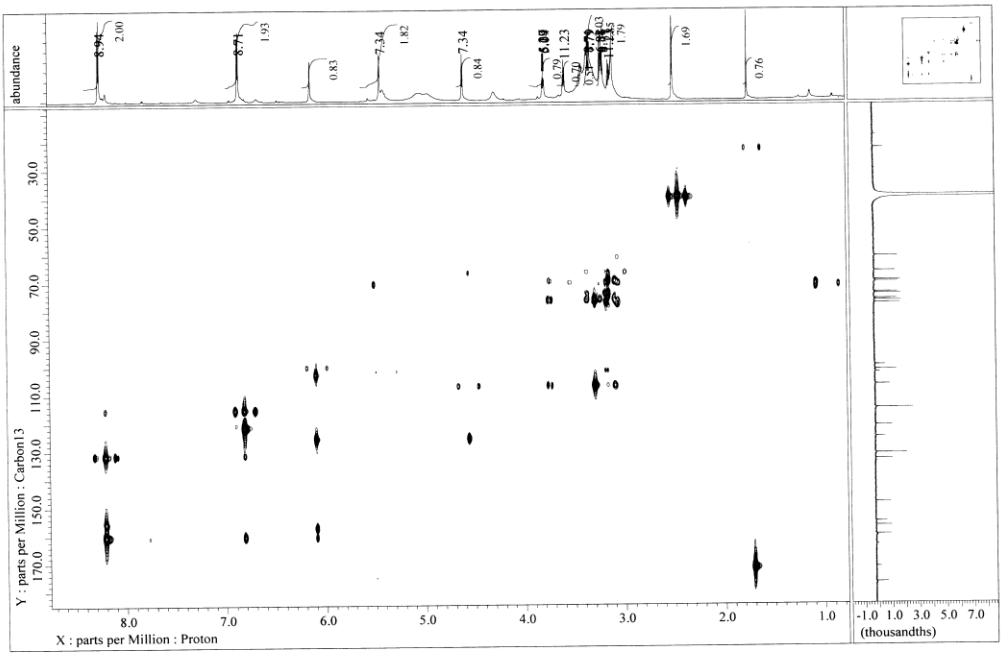

Figure 1-4S. 800 MHz HMBC spectrum of **3** in DMSO-d_6

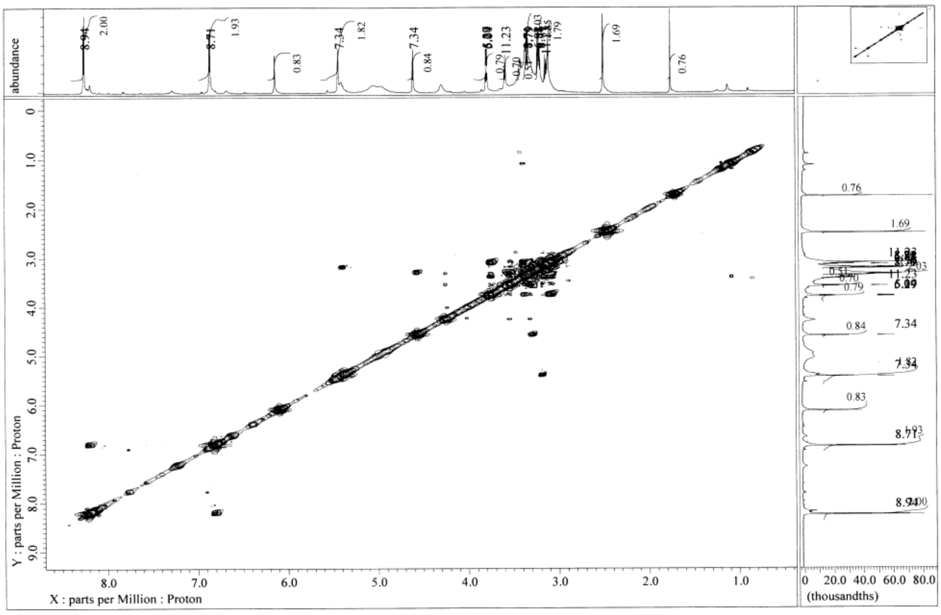

Figure 1-5S. 800 MHz COSY spectrum of **3** in DMSO-d_6

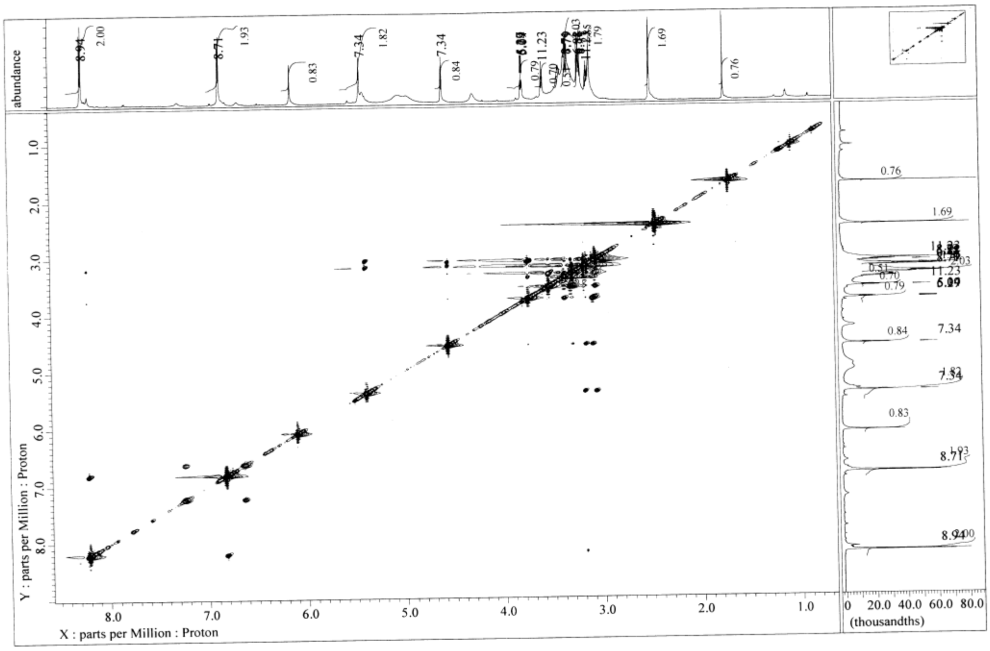

Figure 1-6S. 800 MHz NOESY spectrum of **3** in DMSO-d_6

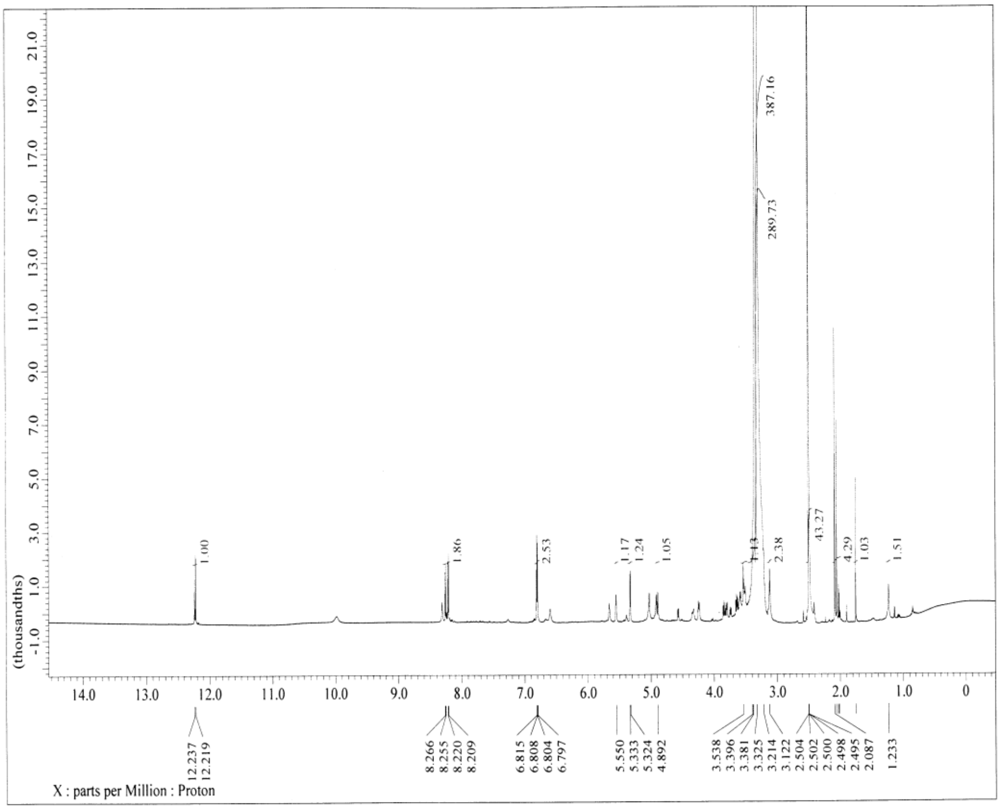

Figure 2-1S. 800 MHz ^{1}H NMR spectrum of **4** in DMSO-d_6

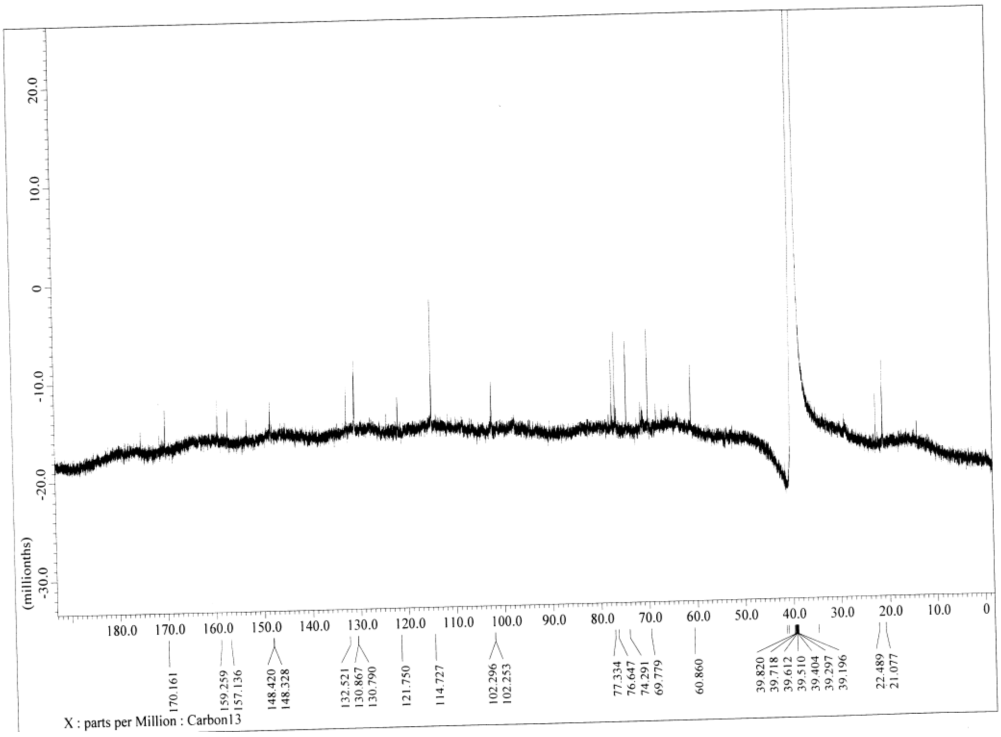

Figure 2-2S. 800 MHz ^{13}C NMR spectrum of **4** in DMSO-d_6

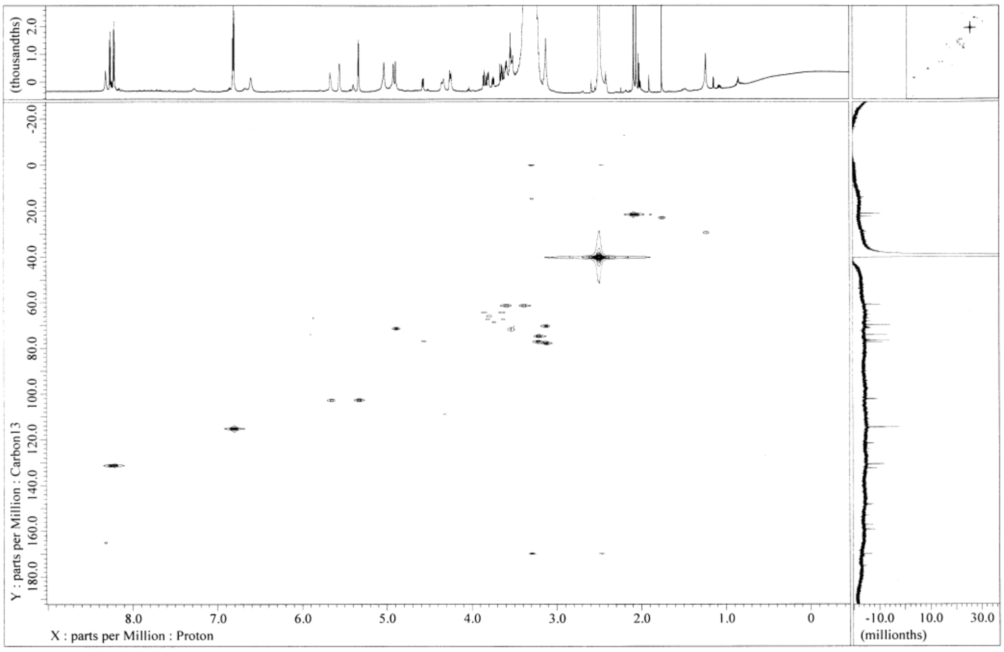

Figure 2-3S. 800 MHz HMQC spectra of **4** in DMSO-d_6

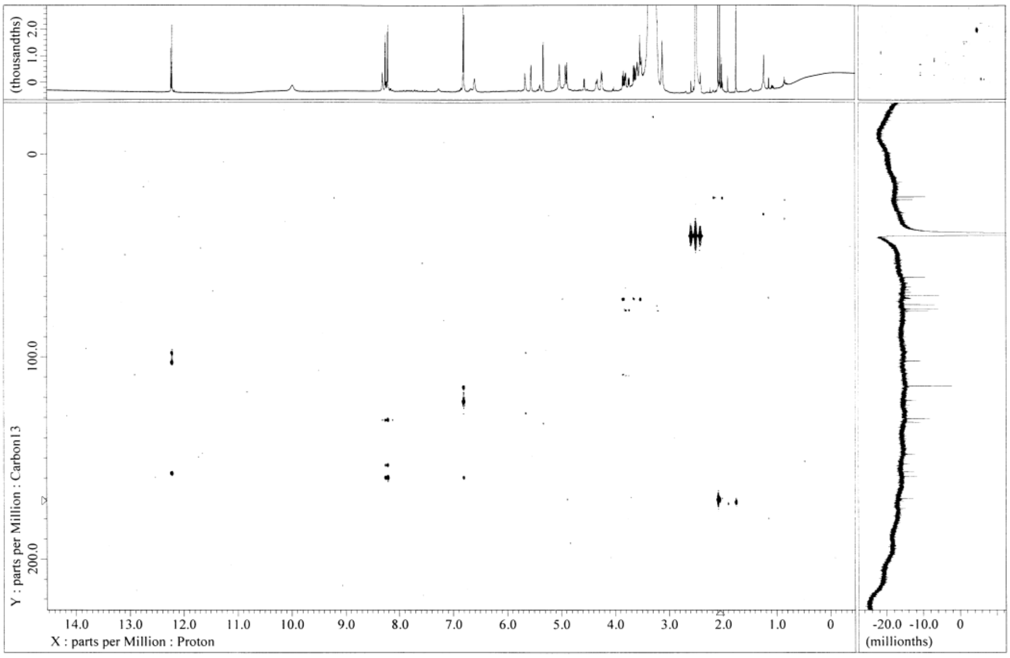

Figure 2-4S. 800 MHz HMBC spectrum of **4** in DMSO-d_6

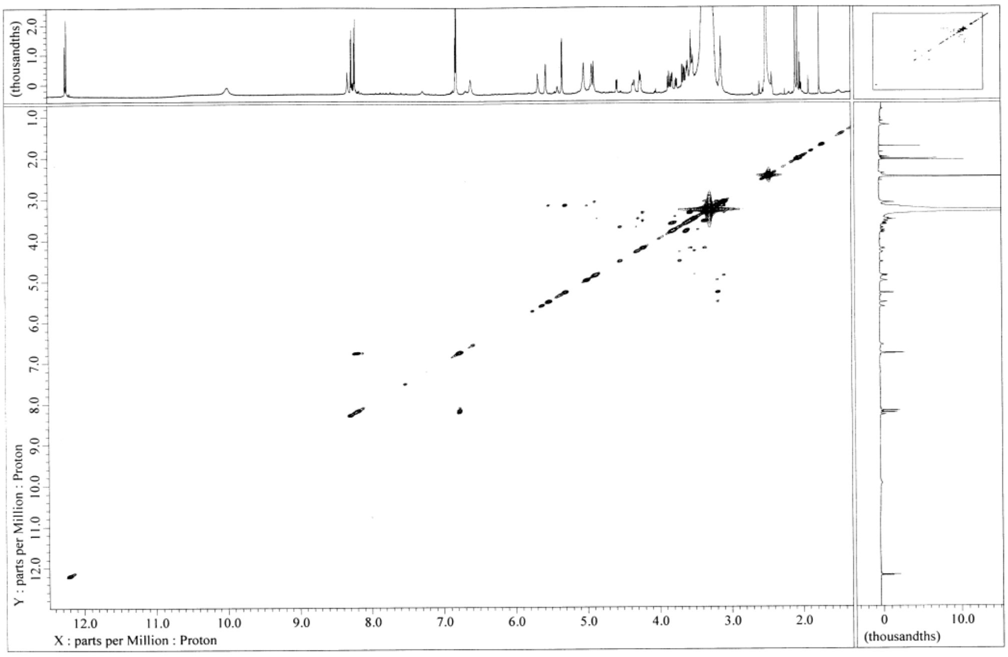

Figure 2-5S. 800 MHz COSY spectrum of **4** in DMSO-d_6

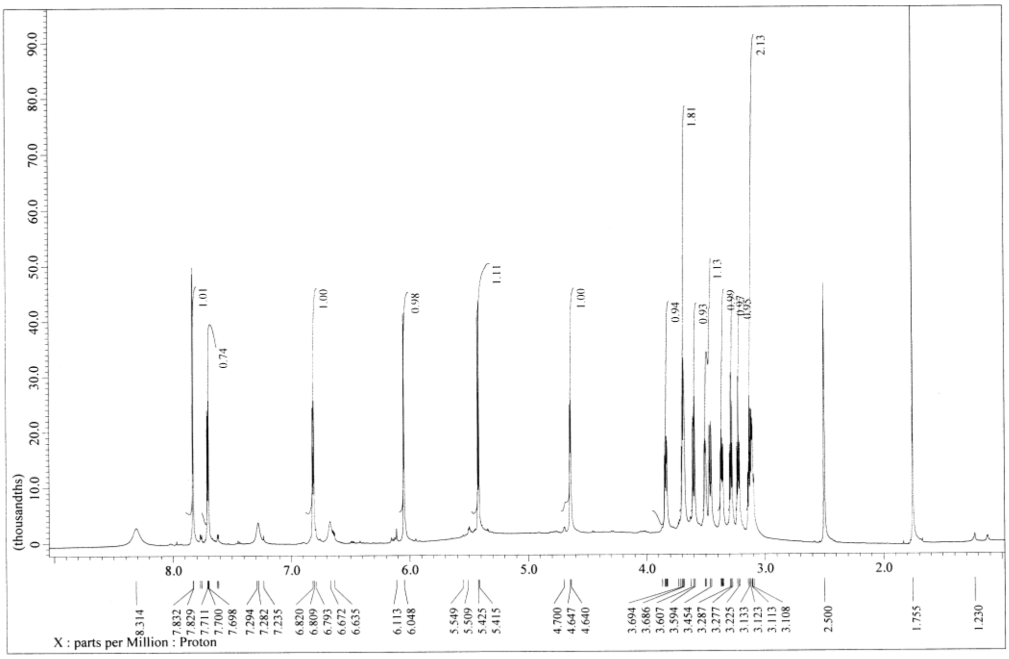

Figure 3-1S. 800 MHz ^{1}H NMR spectrum of **6** in DMSO-d_6

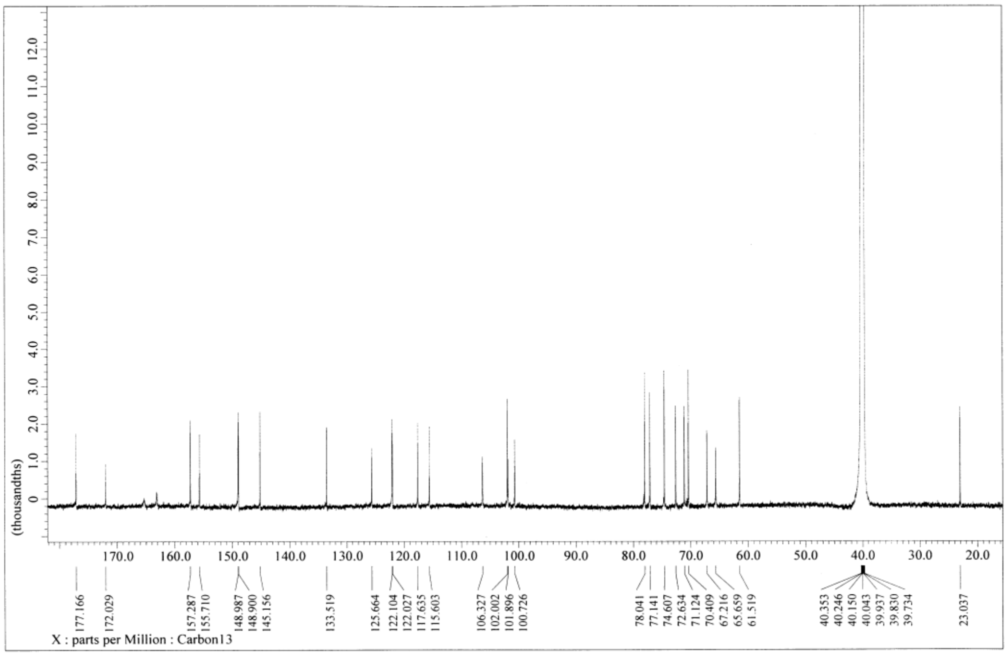

Figure 3-2S. 200 MHz ^{13}C NMR spectrum of **6** in DMSO-d_6

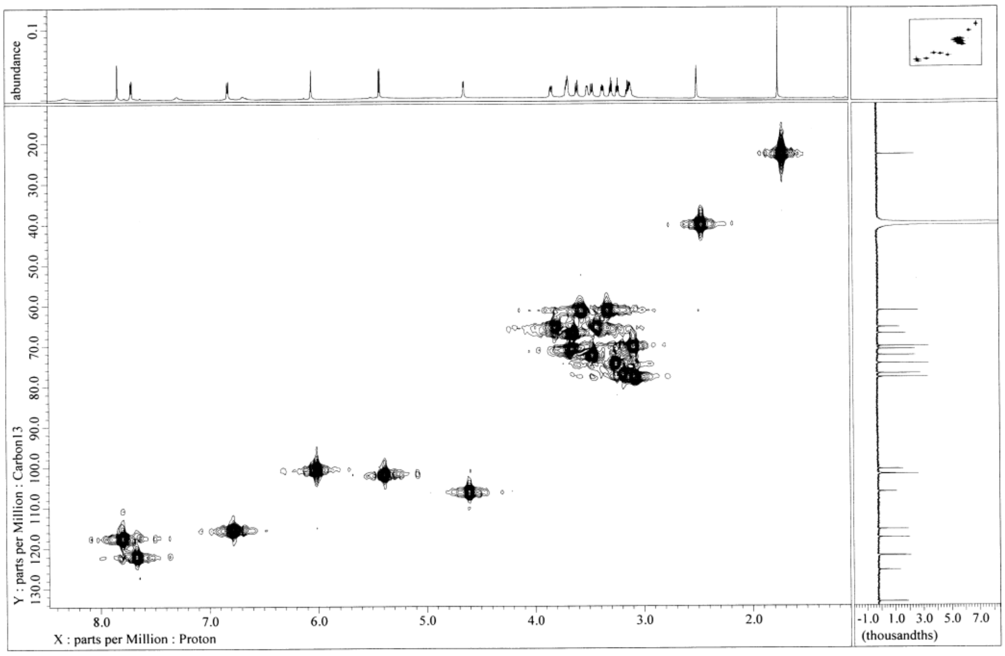

Figure 3-3S. 800 MHz HMQC spectra of **6** in DMSO-d_6

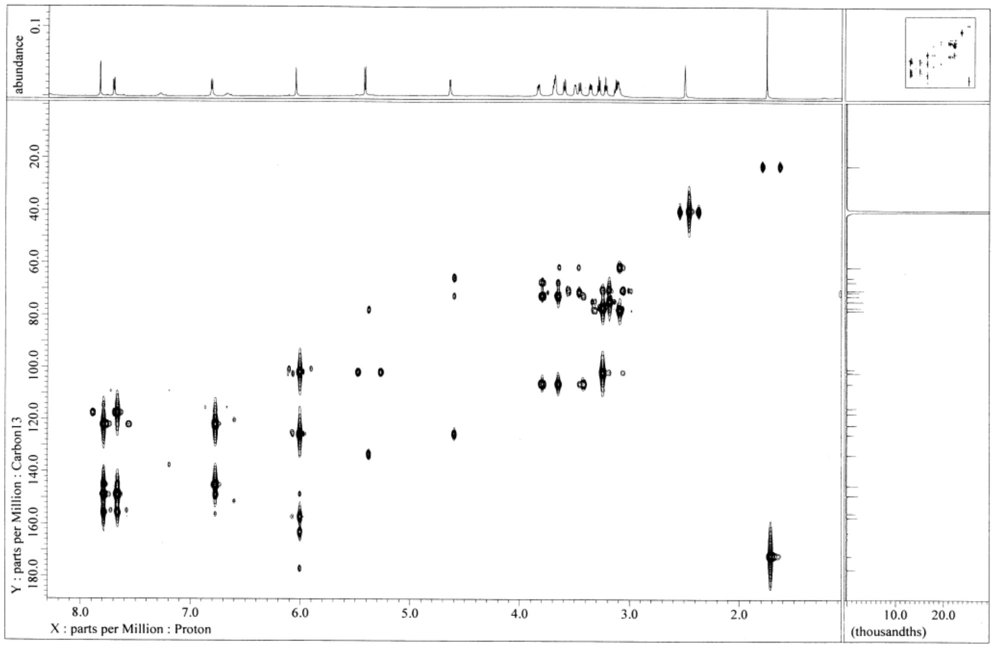

Figure 3-4S. 800 MHz HMBC spectrum of **6** in DMSO-d_6

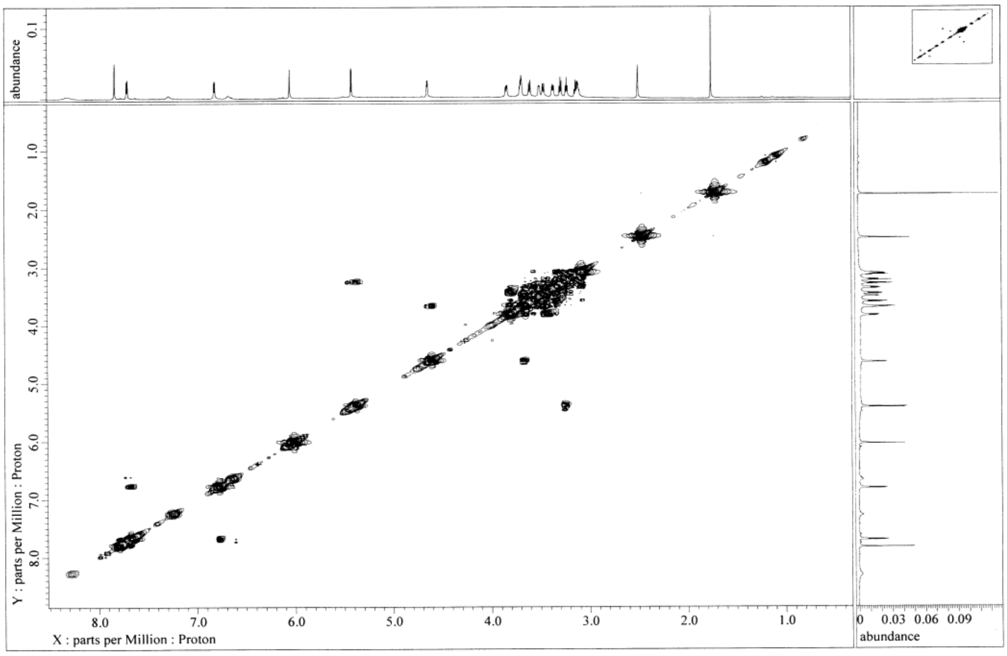

Figure 3-5S. 800 MHz COSY spectrum of **6** in DMSO-d_6

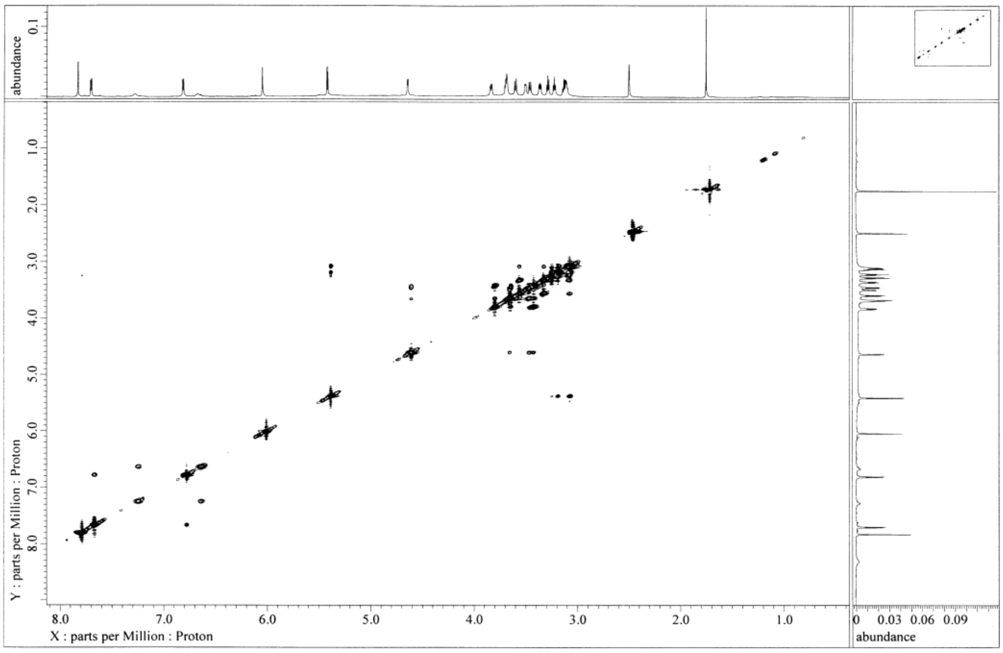

Figure 3-6S. 800 MHz NOESY spectrum of **6** in DMSO-d_6

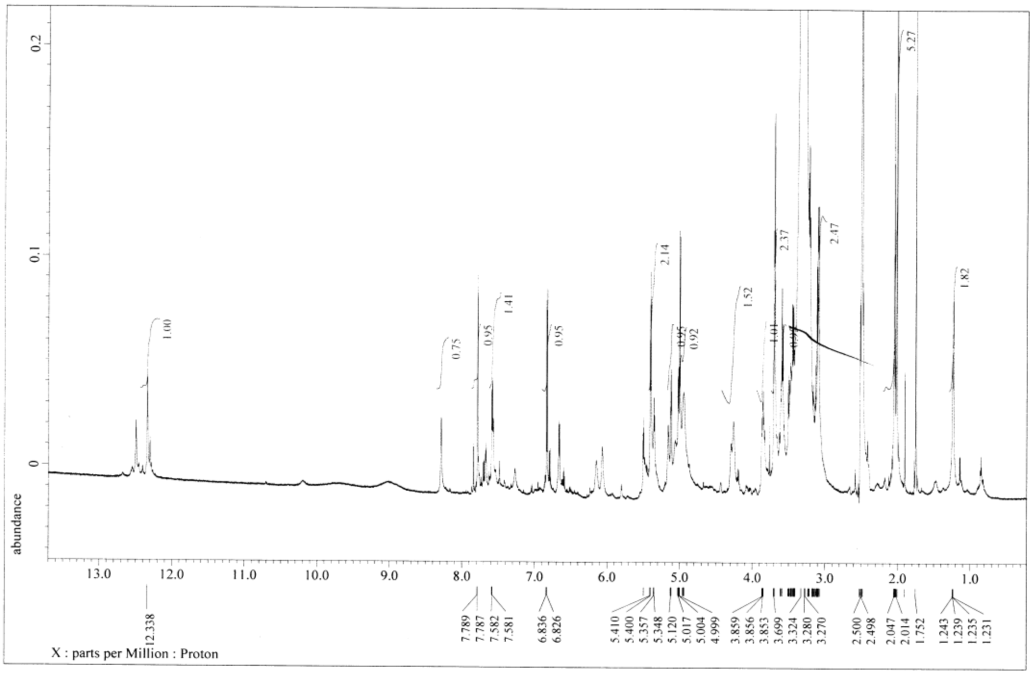

Figure 4-1S. 800 MHz ^{1}H NMR spectrum of **7** in DMSO-d_6

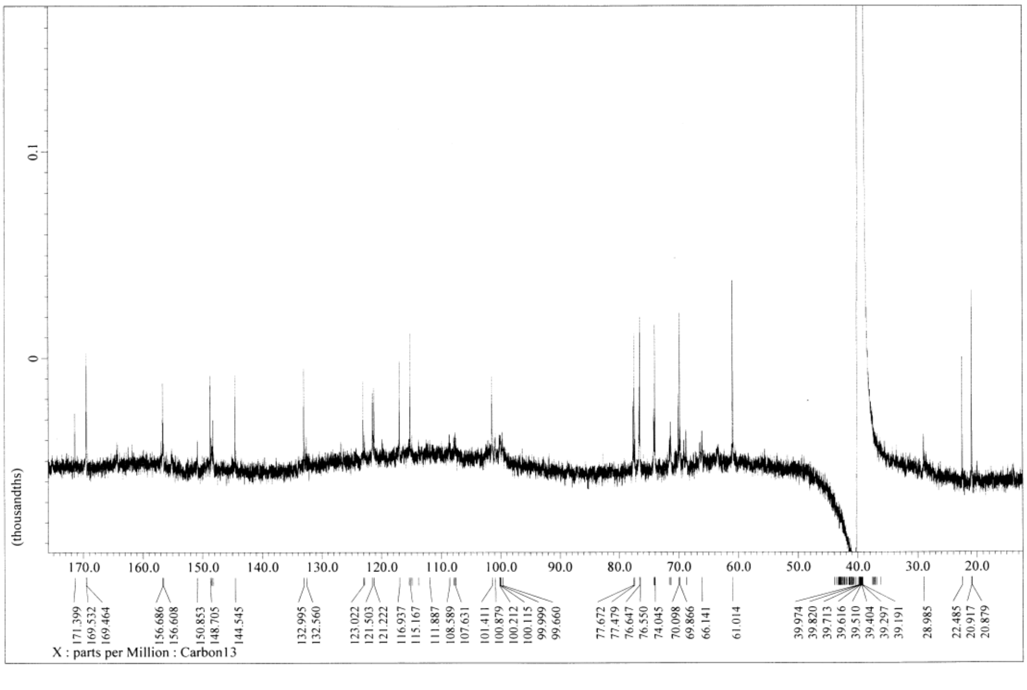

Figure 4-2S. 200 MHz ^{13}C NMR spectrum of **7** in DMSO-d_6

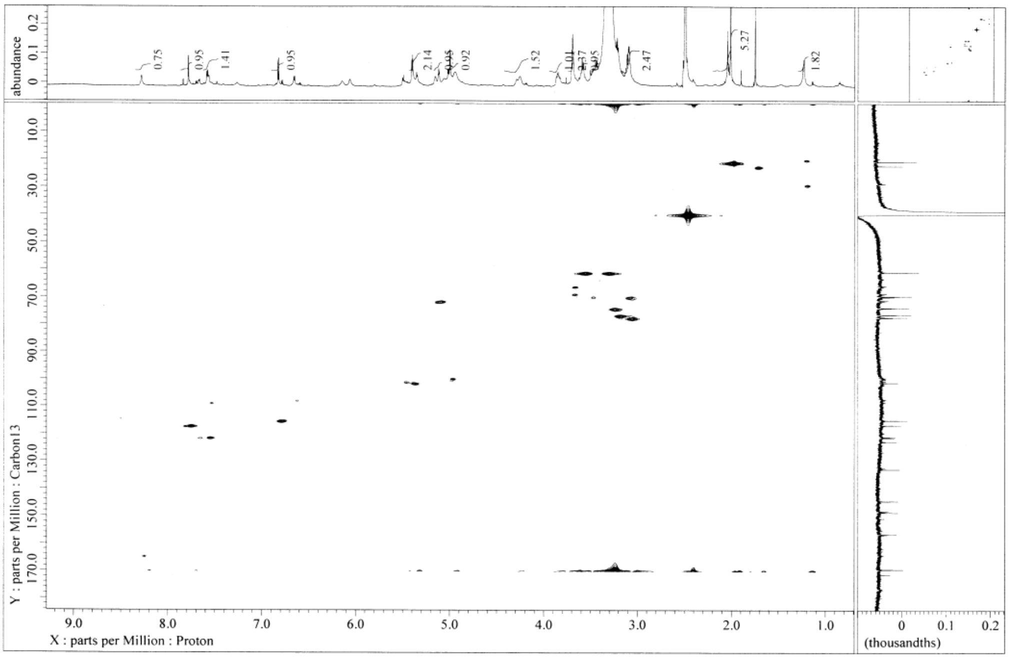

Figure 4-3S. 800 MHz HMQC spectra of **7** in DMSO-d_6

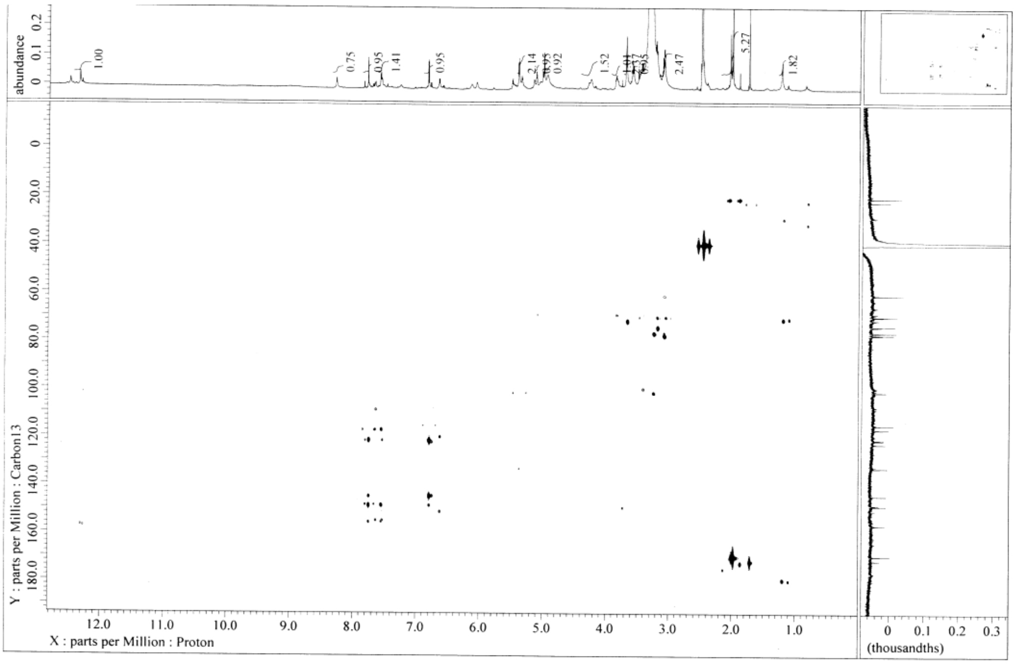

Figure 4-4S. 800 MHz HMBC spectrum of **7** in DMSO-d_6

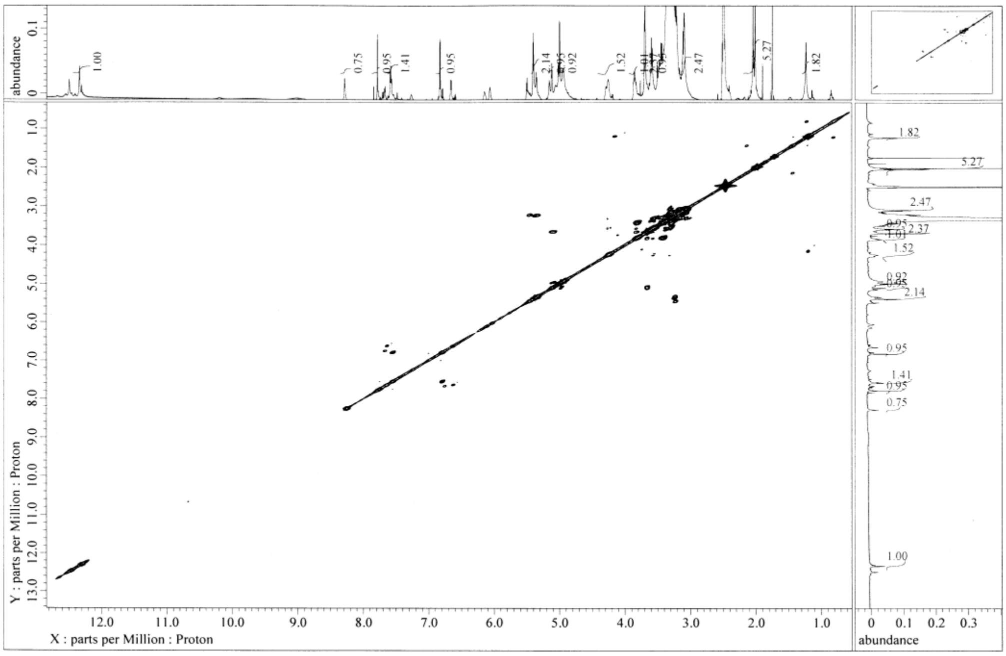

Figure 4-5S. 800 MHz COSY spectrum of **7** in DMSO-d_6

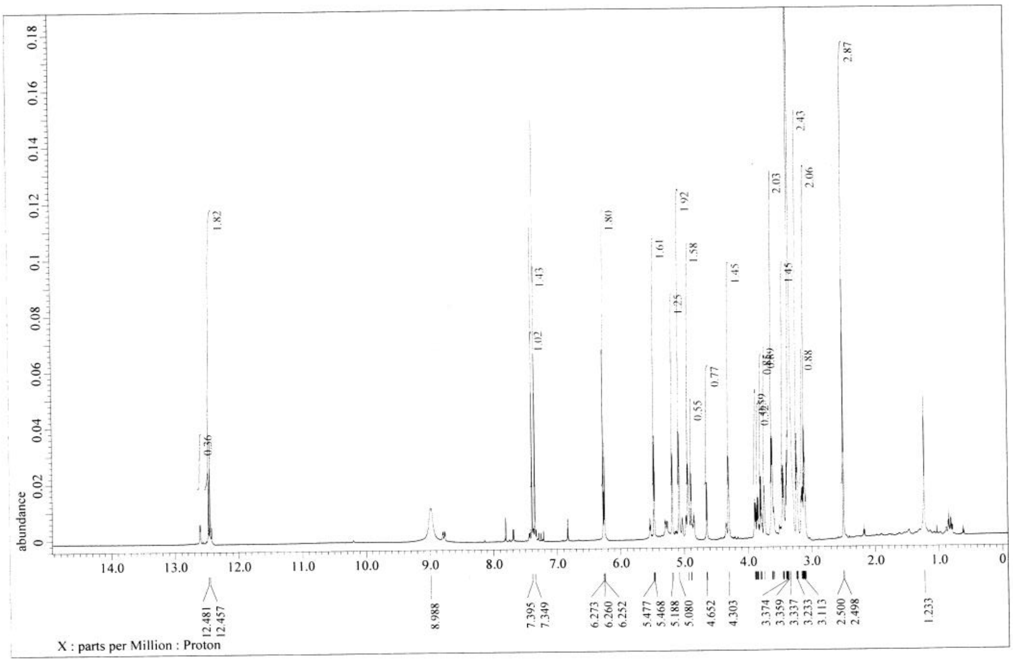

Figure 5-1S. 800 MHz ^{1}H NMR spectrum of **8** in DMSO-d_6

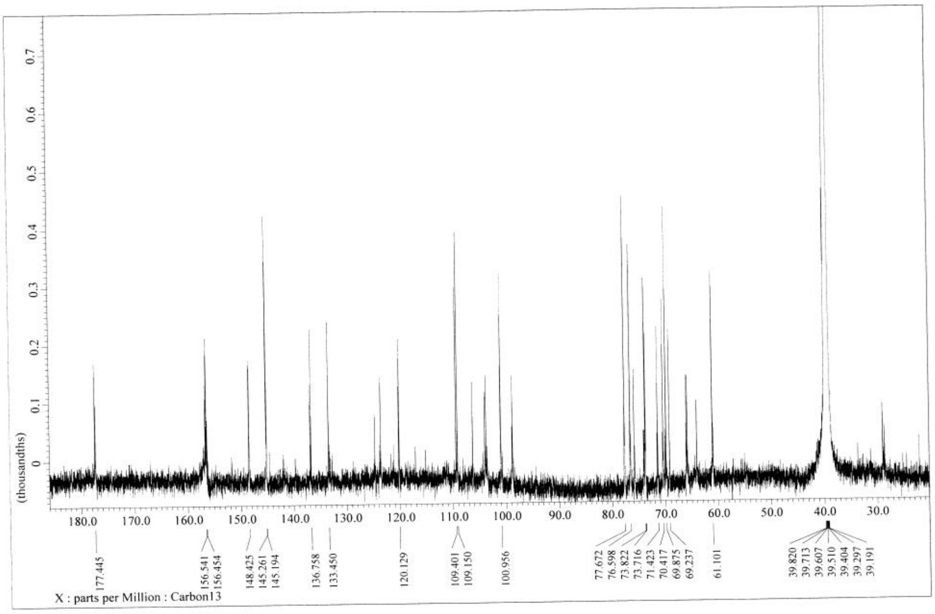

Figure 5-2S. 200 MHz ^{13}C NMR spectrum of **8** in DMSO-d_6

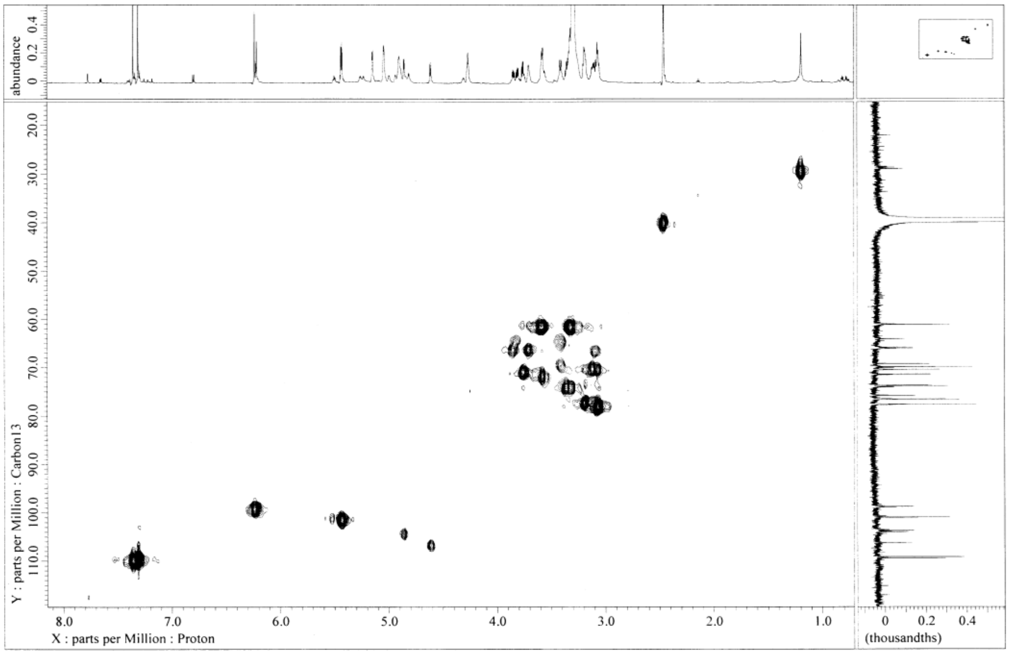

Figure 5-3S. 800 MHz HMQC spectra of **8** in DMSO-d_6

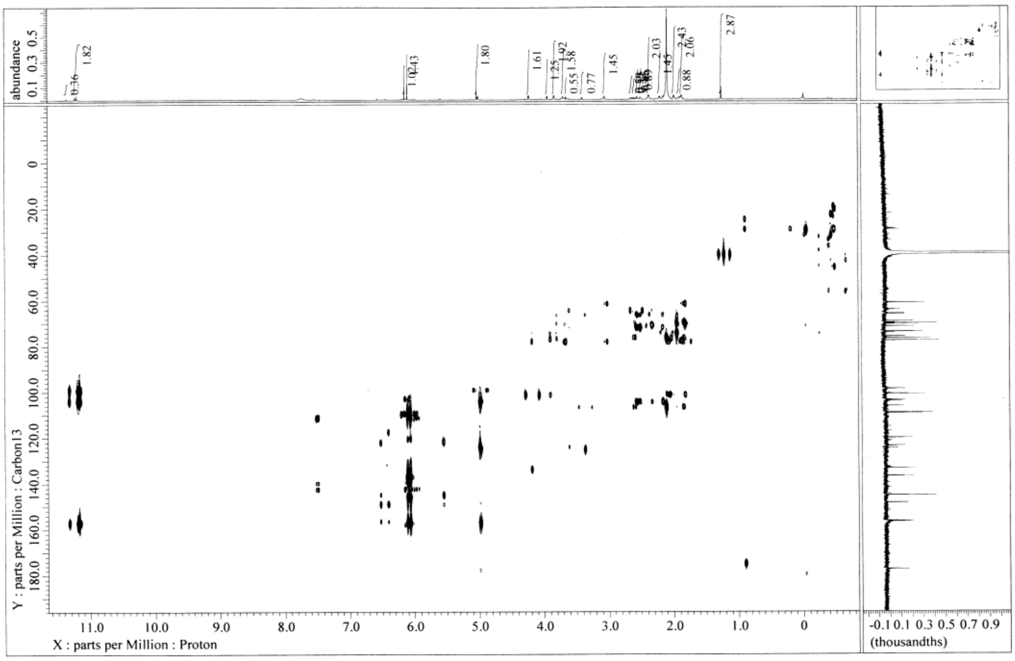

Figure 5-4S. 800 MHz HMBC spectrum of **8** in DMSO-d_6

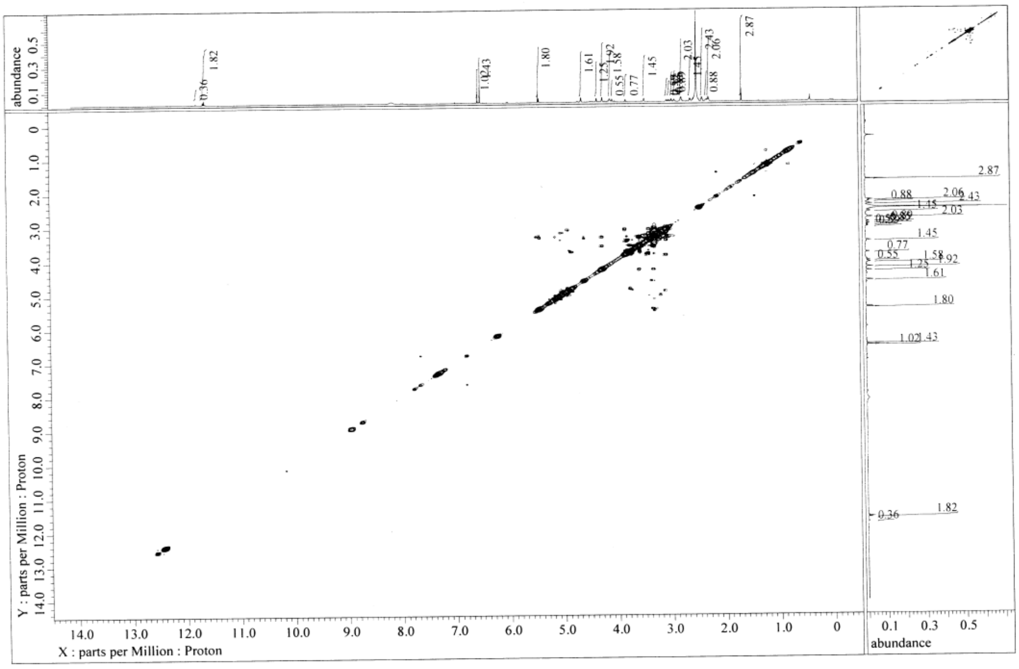

Figure 5-5S. 800 MHz COSY spectrum of **8** in DMSO-d_6

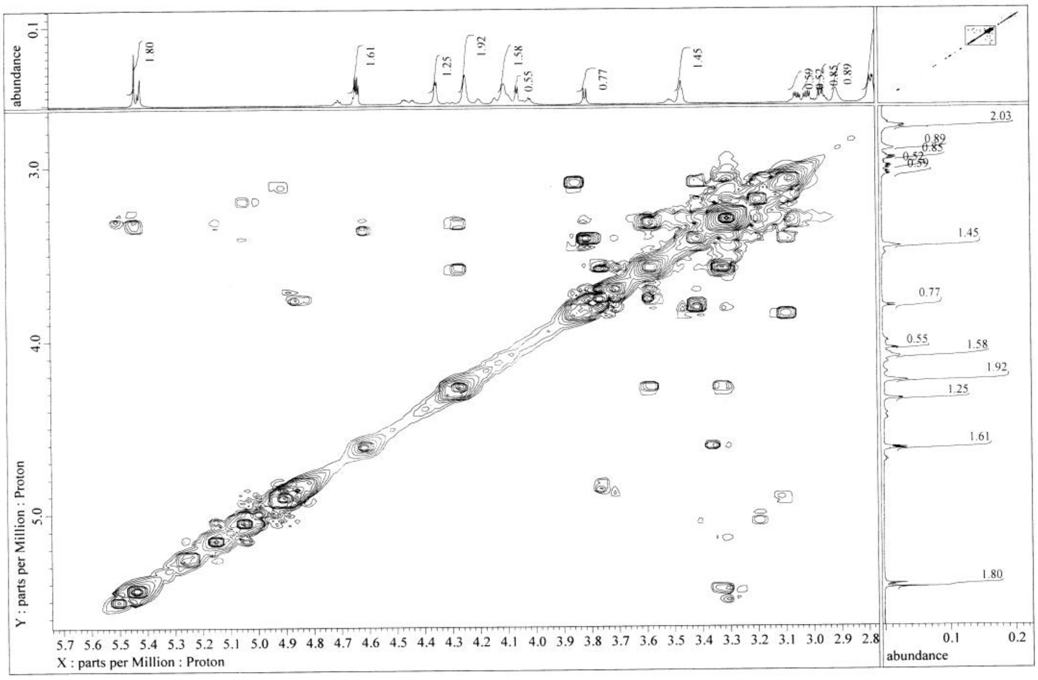

Figure 5-6S. 800 MHz NOESY spectrum of **8** in DMSO-d_6
